# Effect of Syringopicroside Extracted from *Syringa oblata* Lindl on the Biofilm Formation of *Streptococcus suis*

**DOI:** 10.3390/molecules26051295

**Published:** 2021-02-27

**Authors:** Yang Tang, Jingwen Bai, Yu Yang, Xuedong Bai, God’spower Bello-Onaghise, Yaqin Xu, Yanhua Li

**Affiliations:** 1 Department of Applied Chemistry, College of Art and Science, Northeast Agricultural University, Harbin 150030, China; 13834333424@163.com (Y.T.); baijingwen@neau.edu.cn (J.B.); yangyu@neau.edu.cn (Y.Y.); baixuedong456@yeah.net (X.B.); 2 Department of Veterinary Pharmacy, College of Veterinary Medicine, Northeast Agricultural University, Harbin 150030, China; godspower.bello-onaghise@uniben.edu

**Keywords:** *Syringa oblata* Lindl, ultrasound-assisted extraction, syringopicroside, *Streptococcus suis*, biofilm, molecular docking

## Abstract

Syringopicroside is a natural drug with antibacterial activity, which is the main ingredient of *Syringa oblata* Lindl (*S. oblata*). In order to further develop the application of *S. oblata* and evaluate the ability of syringopicroside against *Streptococcus suis* (*S. suis*), this investigation first applied an ultrasonic-assisted method to extract syringopicroside, and then response surface methodology (RSM) was performed to get the optimum condition. Based on RSM analysis, a second-order polynomial equation about the syringopicroside yield and four variables, including ultrasonic power, time, temperature, and liquid-to-solid ratio, was purposed. Through RSM prediction and model verification experiments, the optimum conditions were determined, as follows: ultrasonic time was 63 min, temperature was 60 °C, a liquid-to-solid ratio was set to 63 mL/g, and ultrasonic power was 835 W. Under this condition, a high syringopicroside yield was obtained (3.07 ± 0.13 mg/g), which was not significantly different with a predicated value. After separation and purification by HPD 500 microporous resin, then mass spectrum was applied to identify the main ingredient in aqueous extract. A minimal inhibitory concentration (MIC) assay revealed the value against *S. suis* of syringopicroside was 2.56 µg/µL and syringopicroside with sub-inhibitory concentrations that could effectively inhibit biofilm formation of *S. suis*. Besides, scanning electron microscopy analysis indicated syringopicroside could destroy the multi-layered aggregation structure of *S. suis*. Finally, molecular docking analysis confirmed that syringopicroside was combined with Orfy protein of *S. suis* through hydrogen bonds, hydrophobic interaction, and π-π stacking.

## 1. Introduction

*Syringa oblata* Lindl (*S. oblata*), as a medicine herb, is widely distributed in Northeast and Southwest China [1]. According to the records of Chinese Pharmacopoeia and the compilation of Chinese herbal medicine, one of the pharmacological effects of *S. oblata* was bacteriostasis [2]. Syringopicroside has been proven to be an important antibacterial component in the leaves of *S. oblata* [3]. The antibacterial activity on methicillin-resistant *Staphylococcus aureus* of syringopicroside was identified through in vitro and in vivo experiments [4]. Furthermore, similar activity was presented on *Staphylococcus xylosus* [5]. Zhou et al. reported the antibacterial ability on methicillin-resistant *Staphylococcus aureus* in vivo [6]. As a natural product isolated from *S. oblata*, syringopicroside not only has good anti-bacterial and anti-biofilm properties at a low dose, but also is widely available, easy to extract, less toxic, and less likely to develop drug resistance [7].

At present, simple reflux extraction is mainly used for the utilization of *S. oblata* leave. The disadvantages of this method are long time, high consumption of raw materials, and low extraction yield [7]. Currently, ultrasound-assisted extraction (UAE) has been developed and wildly applied in the extraction of natural products. This technology is not only green and environmentally-friendly, but also is lower cost, shorter time, and higher yield than traditional extraction methods [8,9]. It is worth mentioning that UAE meets the requirements of factory production.

*Streptococcus suis* (*S. suis*) is a zoonotic pathogen that causes serious systemic infections in pigs and humans [10,11]. Many diseases, including pneumonia, septicaemia, and meningitis, can be caused by *S. suis* [12]. Importantly, the main route of human infection with *S. suis* is by contacting with pigs or pork products through skin wounds or consumption of raw pork [13]. During the 1998 and 2005 Chinese epidemics, a total of 240 people was infected with *S. suis*, 53 of whom died [14]. In Thailand in 2010, the incidence proportions of the disease were 6.4/10,000 persons, and the fatality rate was as high as 16.5% [15]. Besides, *S. suis* was predominantly an occupational hazard in North America and Europe [16,17].

In general, the therapy of *S. suis* mainly depends on antibiotics such as erythromycin, azithromycin, tylosin, etc. However, the effect of this treatment is not always as expected due to biofilm. The formation of biofilm is one of the main reasons for the phenomenon in which *S. suis* is hard to cure. More than half of all human microbial infections can be associated with biofilms, indicating biofilm plays a key role in the process of bacterial infection [18]. Biofilm is a bacterial aggregation membrane, which is formed when bacteria adhere to a contact surface, and secrete polysaccharide matrix, fibrin, lipid protein, and so on [19]. The extracellular biofilm matrix serves as a physical barrier. It isolates bacteria from the body’s immune system, and, thereby, shielding the bacteria from natural killer cells, phagocytes, antibodies, lysozymes, sensitized T cells and other immune effects [20,21]. The same effect will work on antibiotics and make antibiotic therapy only kill strains without biofilm. It is noteworthy that drug residues in pig and human, multiple resistance, allergy, and gastrointestinal bleeding and other problems would arise after long-term application of common antibiotics [22]. Therefore, the development and utilization of natural products with antibacterial and antibiofilm properties is particularly urgent [23,24,25].

*S. suis* forms biofilms by binding individual bacterial cells together with capsular polysaccharides. The CPs gene cluster regulates the synthesis of capsular polysaccharide (CPS), which is a vital component of *S. suis*. The cluster contains genes for proteins involved in the synthesis of polysaccharides, such as glycosyltransferase, wzy polymerase (wzy), and wzx flippase (wzx) [26]. In *S. suis* serotype II, the *orfy* gene is located at the head of the gene cluster. Orfy protein, as a transcriptional regulator, belongs to the GntR family and regulates various biological processes [27]. The pervious study proofed that rutin could inhibit biofilm formation by affecting CPS biosynthesis [28].

In a previous study, we found that the aqueous extract of *S. oblata* could effectively inhibit the formation of biofilm in *S. suis* [7]. However, the functional ingredient that played a critical role in this process was not clear. In this study, UAE was used to extract the syringopicroside, and the optimum conditions were determined by using response surface methodology (RSM). The process of separation and enrichment was completed through HPD-500 macroporous resin, and then high-performance liquid chromatograph-tandem mass spectrometry (HPLC-MS) was performed to identify the main active ingredient extracted from *S. oblate* leaves. Moreover, methods of crystal violet staining and scanning electron microscopy (SEM) were used to detect the inhibitory effect of syringopicroside on the formation of biofilm in *S. suis*. Finally, a potential mechanism on the intervention of syringopicroside on biofilm formation of biofilm was inferred via molecular docking.

## 2. Results and Discussion

### 2.1. Effect of Ultrasonic Power on the Extraction of Syringopicroside

**Figure 1:**
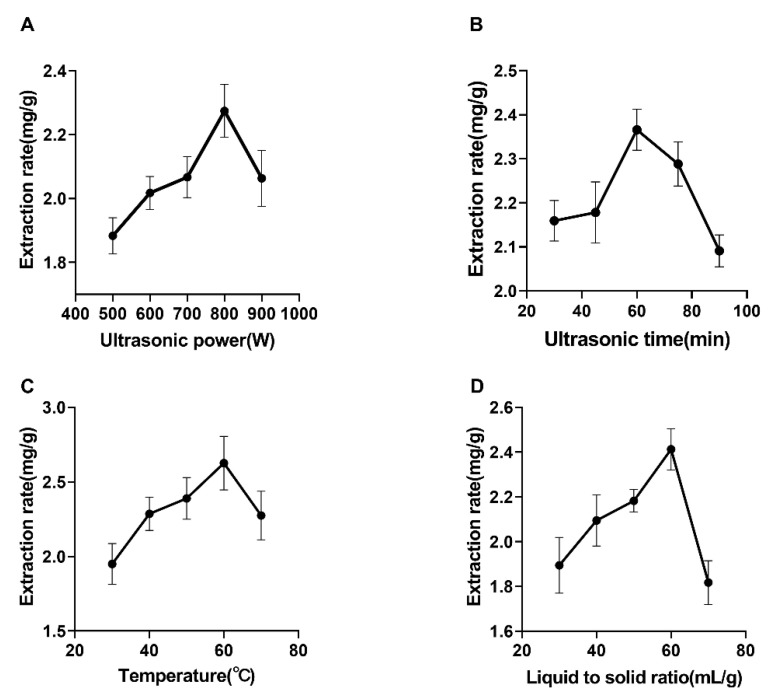
Single-factor experimental results about the ultrasonic-assisted extraction of syringopicroside from *S. oblata* leaves. Different variables were presented in sequence: ultrasonic power (**A**), ultrasonic time (**B**), temperature (**C**), and a liquid-to-solid ratio (**D**). Data was expressed as mean ± standard deviation (*n* = 3).

As shown in Figure 1A, the yield of syringopicroside raised significantly when the power increased from 500 to 800 W. In addition, other conditions were fixed: the temperature was 50 °C, the liquid-to-solid ratio was 50:1 mL/g, and the ultrasonic time was 45 min. The yield declined dramatically once further strengthened power past the point of 800 W. Furthermore, 800 W was the breaking point, while the clove cell wall was destructed effectively because of ultrasonic cavitation, which can make ethanol liquid jets and shock wave [29]. The strengths of cavitation increased with ultrasonic power, and then resulted in the increase of yield before power got to 800 W [30,31]. However, an ultrasonic wave can also let many small bubbles form in medium, which may have an unfavorable effect on intensity of the liquid jet and shock wave [32], and, thereby, decreased the yield of syringopicroside. Therefore, ultrasonic power of 800 W was chosen for syringopicroside extraction.

### 2.2. Effect of Ultrasonic Time on Extraction of Syringopicroside

When the temperature was fixed at 50 °C, the liquid-to-solid ratio was 50:1 mL/g and the ultrasonic power was 800 W, as could be seen from Figure 1B. The yield of syringopicroside increased as the ultrasonic time grew. Nevertheless, as time increased beyond 60 min, the yield declined dramatically. At the initial stage, there was no sufficient time to break the entire plant cell wall by an ultrasonic wave, and the solvent was not able to penetrate the target compound properly. With the increase of ultrasonic time, the wall was sufficiently disintegrated, which boosted permeation of the solvent into cells and facilitated the release of the target compounds into the solvent, resulting in a higher yield of syringopicroside [33]. However, the yield of syringopicroside began to degrade and the structure was destroyed due to excessive use of an ultrasonic wave. This result was similar to that of Ponmurugan et al. [29]. Based on the above result, the ultrasonic time of 60 min was selected for the following experiments.

### 2.3. Effect of Temperature on the Extraction of Syringopicroside

Observed from Figure 1C, the yield of syringopicroside increased with the gradual increase in temperature, while the yield began to decrease when the temperature was increased beyond 60 °C. In addition, the ultrasonic power was set to 800 W, the liquid-to-solid ratio was 50:1 mL/g and the ultrasonic time was 45 min. A similar phenomenon occurred during the extraction of flavonoid from *Morus alba* L. leaves [34] and *Sophora flavescens* [35]. Cavitation and thermal effects play a critical role in this kind of experiment [36]. The thermal effect can enhance the destruction of the cell matrix, and further improve the mass transfer rate of the target compound and facilitate the intracellular parts dissolved into the solvent [37]. At a low temperature (30 °C), the thermal effect was not noticeable, and the low yield was obtained. The effect of a thermal effect enhanced with the increase of temperature, which led to a relatively high yield. However, beyond 60 °C, the role of a thermal effect was superior to cavitation, leading to an unfavorable effect on cavitation [38]. At the same time, increasing the extraction temperature would cause the degradation of the target compound and accelerate the evaporation of the solvent [39,40]. Through the analysis above, 60 °C was found to be the suitable temperature for the next phase of the experiments.

### 2.4. Effect of a Liquid-to-Solid Ratio on The Extraction of Syringopicroside

The conditions including ultrasonic power, time, and temperature were fixed as 800 W, 45 min, and 50 °C, respectively. It could be seen that the yield of syringopicroside was significantly improved as the liquid-to-solid ratio increased from 30 to 60 mL/g (Figure 1D). However, the yield decreased clearly when the ratio was increased further. Similar results have been reported by Abulimiti Yili et al. [41]. At a low liquid-to-solid ratio, the power tended to cluster together, inhibit solvent getting in, and cause a low yield [42]. At the initial stage, the high concentration difference that existed between the inner of the plant cell and the exterior solvent, promoting the dissolution of a target compound in solvent. Nevertheless, due to the increment of dissolved impurities such as protein, polysaccharides can impede the target compound that dissolved into extraction solvent [29]. Moreover, the use of a large amount of volume solvent was costly. Based on this result, a suitable liquid-to-solid ratio of 60 mL/g was selected for the subsequent experiments.

### 2.5. Optimization of the UAE Process with RSM

#### 2.5.1. Statistical Analysis and Model Fitting

As could be seen from Table 1, the yield of syringopicroside ranged from 1.83 to 3.16 mg/g with the changes in different variables. According to the multiple regression analysis, a second-order polynomial equation about a predicted response for syringopicroside yield was obtained. The equation was as follows (1):

(1) $$Y=3.02+0.11X_{1}+0.28X_{3}+0.18X_{4}-0.12X_{2}X_{4}-0.20X_{1}^{2}-0.15X_{2}^{2}-0.44X_{3}^{2}-0.23X_{4}^{2}$$

where Y represents the yield of syringopicroside, $X_{1}$, $X_{2}$, $X_{3}$, and $X_{4}$ mean variables of ultrasonic time, temperature, liquid-to-solid ratio, and ultrasonic power, respectively.

The result of the ANOVA was presented in Table 2. First, the “*F*-value” of the model was 15.04 with a low probability (*p* < 0.0001), indicating the significance of the model [43]. The model coefficient of determination (*R*^2^) was 0.9493, showing a strong correlation between the anticipated and experimental values [31]. Moreover, the adjusted coefficient of determination (*R*^2^*_adj_*, 0.8986) confirmed the accuracy of the model [44]. A low coefficient of variance value (*C.V*%, 4.20) suggested that the experimental values and predicted values had little deviations, and indicated higher precision and reliability of the model [45]. The lack of fit (*F* = 1.80, *p* = 0.3007) was non-significant, signifying the model was in line with previous experimental results, showing adequate accuracy of the model. According to Table 1, the linear coefficients of *X*_1_, *X*_3_, and *X*_4_, the quadratic coefficients of *X*_1_^2^, *X*_2_^2^, *X*_3_^2^, and *X*_4_^2^, and *X*_2_*X*_4_ were significant (*p* < 0.05), suggesting these terms had a significant effect on the response. However, *X*_2_, *X*_1_*X*_2_, *X*_1_*X*_3_, *X*_1_*X*_4_, *X*_2_*X*_3_, and *X*_3_*X*_4_ were non-significant model terms because their *p*-value were above 0.05. Consequently, only the terms with a significant coefficient were applied for model building. At the same time, this model could exhibit the maximum value because of the quadratic coefficients of *X*_1_^2^, *X*_2_^2^, *X*_3_^2^, and *X*_4_^2^ were negative. By comparing the liner coefficient values, the order of the variables impacting on the yield of syringopicroside was: *X*_3_ > *X*_4_ > *X*_1_ > *X*_2_. Thus, the liquid-to-solid ratio showed the most significant effect on the yield of syringopicroside. Next was ultrasonic power, time, and temperature.

#### 2.5.2. Model Adequacy

**Figure 2:**
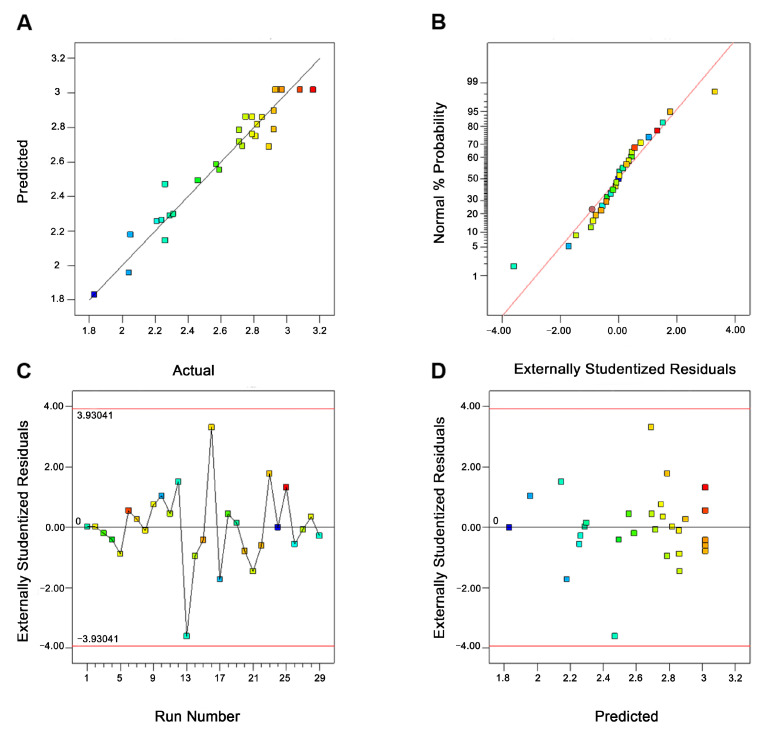
Diagnostic plots for the response surface model adequacy. (**A**) Plot of predicted versus the actual values. (**B**) The normal percent Probability plot. (**C**) Plot of internally studentized residuals versus the formula predicted. (**D**) Plot of internally studentized residuals versus the experimental run.

From Figure 2A, the data points were close to the straight line, indicating a strong correlation existed between the predicted and the experimental values. The plot of normal percent probability was shown in Figure 2B. It could be seen that the distribution of the data was closed to a straight line, signifying both the normal distribution and the variance had no significant deviation. Figure 2C,D revealed that all data points were placed in a reasonable range within the red line. These results suggested that this model built by RSM was accurate and reliable to interpret the experiment involving the UAE for the syringopicroside from *S. oblata*.

#### 2.5.3. Influence of Variables upon Syringopicroside Yield

**Figure 3:**
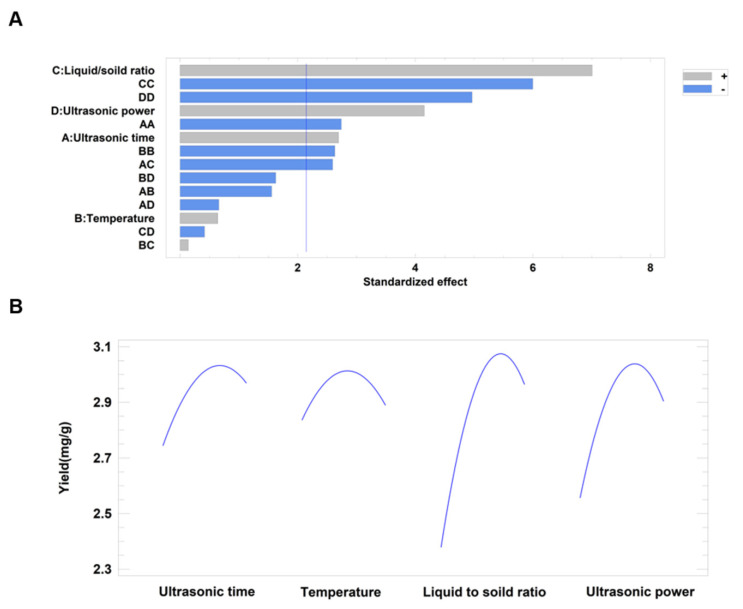
Standardized Pareto chart for efficiency (**A**) and effects of four parameters variation on syringopicroside yield (**B**). The different color of the bar represents the positive or negative effects of the variables on the response value. The blue vertical line means a statistically significance with the 95% confidence level.

The standardized effect of variables on syringopicroside yield was presented in the Pareto chart (Figure 3A). The blue vertical line indicated a 95% confidence level. Every bar beyond this line can be considered as having a significant effect on the yield of syringopicroside. The positive (+) sign meant a positive role in syringopicroside extraction and the negative (−) sign meant a negative role. The length of the bar was correspondent to the statistical value. As could be seen in Figure 3A, the effect of a liquid-to-solid ratio was the most significant one among four variables on the process of syringopicroside extraction. The yield would increase with the growth of these four variables. The same result could be obtained in Figure 3B. It could be found that the variation of the liquid-to-solid ratio had the most significant influence on yield, which was followed by the ultrasonic power, ultrasonic time, and temperature.

**Figure 4:**
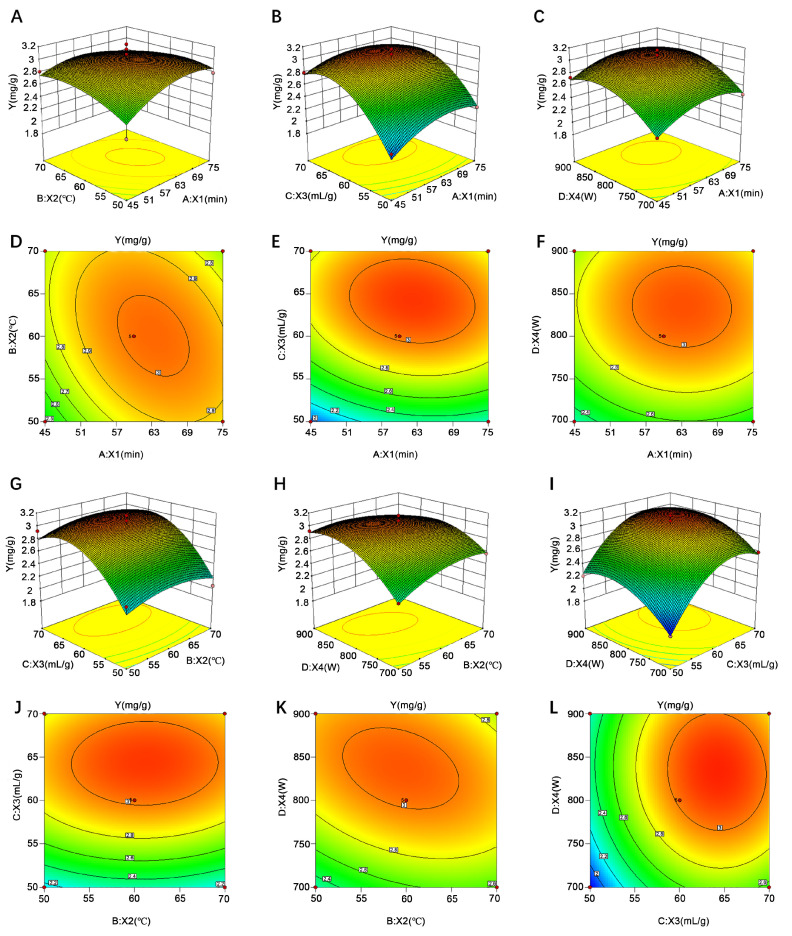
Response surface plot of variables interaction on influence of syringopicroside yield. Variables were such as ultrasonic time (*X*_1_, min), temperature (*X*_2_, °C), liquid-to-solid ratio (*X*_3_, mL/g), and ultrasonic power (*X*_4_, W). (**A**–**C**) represents the interaction between *X*_1_ and *X*_2_, *X*_1_ and *X*_3_, and *X*_1_ and *X*_4_ on effect of yield of syringopicroside, respectively. (**G**–**I**) shows the syringopicroside yield as related to *X*_2_ and *X*_3_, *X*_2_ and *X*_4_, *X*_3_, and *X*_4_. Graphic of (**D**–**F**), (**J**–**L**) was a contour plot that, corresponding to the above response surface plot, reflecting every two variables’ impact on a syringopicroside yield.

By analyzing experimental data, the three-dimensional response surface and contour plots were obtained. The effect of the interaction among the variables on yield was shown in Figure 4. As presented in Figure 4A,D, the yield increased initially and decreased slightly with the increase of ultrasonic time from 45 to 75 min and temperature from 50 to 70 °C. The effect of the interaction between a liquid-to-solid ratio and ultrasonic time was shown in Figure 4B,E. With the increase of liquid to solid from 50:1 to 70:1 mL/g and ultrasonic time from 45 to 75 min, the yield increased to a maximum value and then decreased slightly. The yield was relatively sensitive to the variation of a liquid-to-solid ratio due to the steepness sloop [46]. As shown in Figure 4C,F, the maximum yield was observed near the midpoint region. Furthermore, the slope of ultrasonic power was steeper than that of time, indicating its significant influence on the yield. At the investigation of the interaction of the liquid-to-solid ratio and temperature, a similar tendency between ultrasonic time and the liquid-to-solid ratio was observed (Figure 4G,J). The interaction effect of ultrasonic power and temperature was presented as a visual form in Figure 4H,K. It could be found that, with an increase in the level of ultrasonic power from 700 to 900 W and temperature from 50 to 70 °C, there was a gradual increase in the yield of syringopicroside, whereas the yield had a small decline once the two variables increased unceasingly. Observed from Figure 4I,L, an optimum yield of syringopicroside was obtained when the liquid-to-solid ratio and ultrasonic power was close to 60:1 mL/g and 800 W, respectively. The slopes of the two variables were steep, which revealed that the liquid-to-solid ratio and ultrasonic power had significant impacts on the yield. These results were in tandem with ANOVA analysis (Table 1).

#### 2.5.4. Verification of the Model

Through the analysis of the experimental data, the optimum conditions for UAE were as follows: ultrasonic time was 63.57 min, temperature was 60.35 °C, a liquid-to-solid ratio was 63.17 mL/g, and ultrasonic power was 835.45 W. Under this condition, the predicted yield of syringopicroside was 3.10 mg/g. According to the feasibility of the experiment, the parameters were adjusted as follows: ultrasonic time was 63 min, temperature was 60 °C, the liquid-to-solid ratio was set to 63 mL/g, and ultrasonic power was 835 W. Based on the modified conditions, the yield of syringopicroside was 3.07 ± 0.13 mg/g. There was no significant difference between the predicted value and the experimental value, indicating high accuracy and effectiveness of the model.

### 2.6. Separation and Identification of Syringopicroside

**Figure 5:**
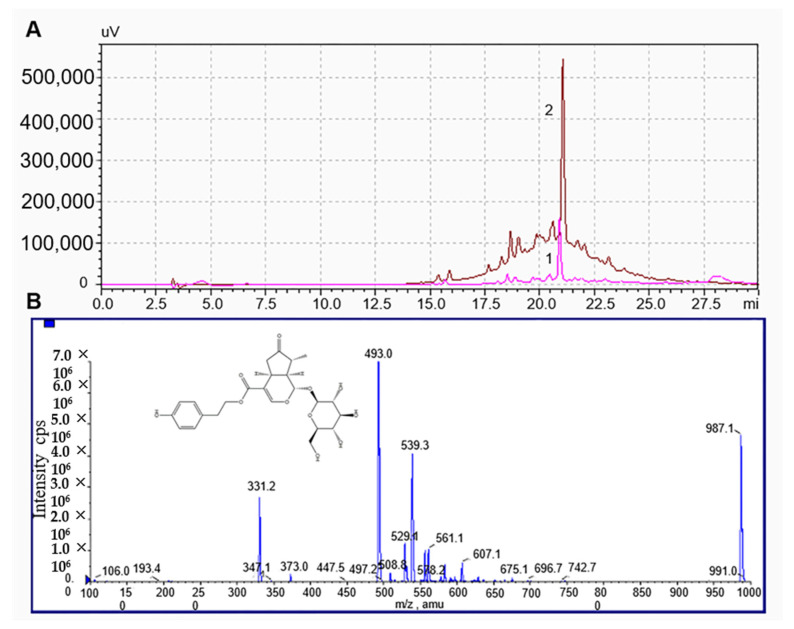
Liquid chromatography of syringopicroside standard (Line 1) and purified sample (Line 2) (**A**) and mass spectra of a purified sample (**B**).

After the treatment with HPD-500 macroporous resin and ethyl acetate, the purity of syringopicroside was improved from 25.61% ± 0.53% to 78.53% ± 0.24%. The liquid chromatograms of a syringopicroside standard and purified sample were shown in Figure 5. As could be seen in Figure 5A, there was a maximum absorption at 21 min for a syringopicroside standard (Line 1). For the purified sample (Line 2), its maximum absorption peak time was the same as that of the syringopicroside standard, indicating the main ingredient of a purified sample was syringopicroside. A molecular cation was detected mainly in the [M − H]^−^ form (Figure 5B). The highest peak, producing a negative molecular ion at *m*/*z* of 493.0 Da, was attributed to syringopicroside. This result was consistent with the previous report about syringopicroside mass data [47]. Therefore, syringopicroside was determined as the main ingredient in *S. oblata* extract.

### 2.7. Effect of Syringopicroside on Biofilm Formation of S. suis

#### 2.7.1. Minimum Inhibition Concentration (MIC) and Crystal Violet Staining Assay

**Figure 6:**
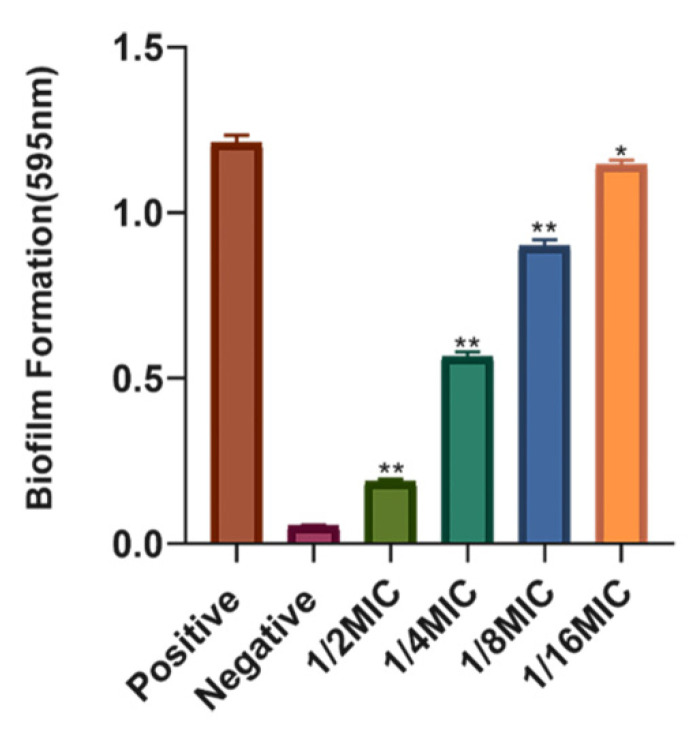
The influence of syringopicroside on *Streptococcus suis* biofilm formation. The data were expressed as means ± SD. ** *p* < 0.01, * *p* < 0.05.

At a series of concentration experiments, *S. suis* could grow until the concentration of syringopicroside was diluted to 1.28 μg·μL^−1^. Therefore, the MIC of syringopicroside on *S. suis* was 2.56 μg·μL^−1^. The MIC of emodin against *S. suis* was 1 mg·mL^−1^ [48], which was clearly higher than that of syringopicroside. Therefore, the syringopicroside extracted in this study could effectively inhibit the growth of *S. suis* by a minimal concentration. The previous study also found that syringopicroside as the main active fraction of *Syringae Folium* displayed a good inhibitory effect on methicillin-resistant *Staphylococcus aureus* (MRSA) with MIC of 1.28 mg/mL. Moreover, syringopicroside was effective in treating MRSA-infected mice, resulting in a significant increase in the survival rate (from 42.8% to 92.8%) [4]. As indicated in Figure 6, sub-inhibitory concentrations of syringopicroside (1/2, 1/4, 1/8, and 1/16 MIC) significantly inhibited the biofilm formation of *S. suis* (*p* < 0.05) in a concentration-dependent manner. Above all, syringopicroside showed good potential in both antibacterial and biofilm inhibition properties.

#### 2.7.2. SEM Observation

**Figure 7:**
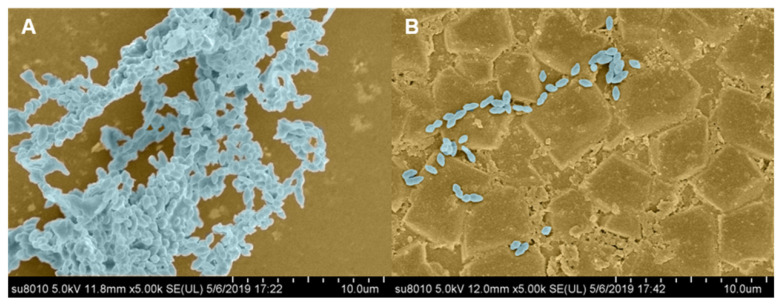
Scanning electron micrographs of *Staphylococcus suis* biofilm formation in the condition of no addition of syringopicroside (**A**) or treated with ½ minimum inhibition concentration (MIC) of syringopicroside (**B**) in Todd-Hewitt Broth (THB) culture medium.

Observed from Figure 7A, the bacteria gathered together to form a multi-level three-dimensional structure, namely, biofilm. After treatment with one-half of MIC of (1.28 μg·mL^−1^) syringopicroside, only a few bacteria were observed with scattered distribution, and there was no multi-level three-dimensional structure formed (Figure 7B). Comparing these two graphs, syringopicroside could significantly intervene with the formation of biofilm in *S. suis*. Thus, it can be inferred that syringopicroside has potential in the treatment of diseases caused by *S. suis*.

### 2.8. Molecular Docking

**Figure 8:**
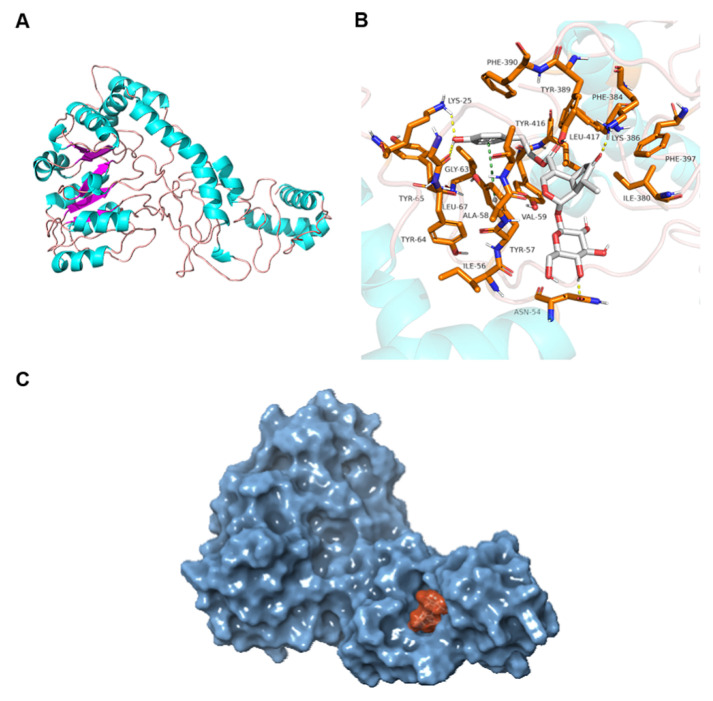
The structure of Orfy protein was shown in (**A**). The binding mode of syringopicroside combing with Orfy protein (**B**). The white sticks represent the ligand and the orange sticks represent residues. The yellow dotted line represent the hydrogen bond. The whole surface graphic of the ligand-protein complex (**C**). The orange part is syringopicroside and the blue part is Orfy protein.

Modeling protein structure was shown in Figure 8A. It could be found that alpha helix, *β*-folding, *β*-folding, and an irregular curl existed in the protein’s second structure. The blue part was an alpha helix and the pink represented an irregular curl, while the purple was *β*-folding. The quality assessment of the optimized Orfy protein was shown in Figure S2.

As could be seen in Figure 8B, the syringopicroside could form hydrogen bonds with several residues of Orfy protein including LYS25, ASN54, GLY63, and LYS386. Furthermore, there existed a hydrophobic interaction between syringopicroside and residues such as ILE56, TYR57, ALA58, VAL59, TYR64, TYR65, LEU67, ILE380, PHE384, TYR389, HE390, TYR416, and LEU417, and there was a formation of π-π stacking between syringopicroside and residue TYR57 (Table 3). Importantly, the residue of TYR57 played an essential role in the combination process due to the multiple forces which existed between syringopicroside and the residue. The formation of this force plays a vital role on the combination process. These hydrophobic contacts could release the water molecules present in a hydrophobic area, leading to an increase in the entropy of the system, which, in turn, reduces the change of free energy, and improves ligand affinity. From the observation of a surface format (Figure 8C), syringopicroside could fit into the cave-like active site of Orfy protein very well and form a complex. This result was consistent with the induced fit theory. In conclusion, syringopicroside could be well combined with the Orfy protein of *S. suis*. The binding affinity was −9.182 kcal/mol. The combination of syringopicroside and Orfy protein would change the conformation of Orfy protein and influence the expression of *cps* gene clusters, which, in turn, led to a decrease in the expression of the proteins related to the synthesis of a polysaccharide. This result was consistent with the previous study. The expression of CPS6J and CPS28H protein was down-regulated after treatment with an aqueous extract [7]. The synthesis of capsular polysaccharide decreased due to the decrease in the expression of related proteins, leading to the inhibition of the formation of biofilm by *S. suis*. Certainly, we inferred the interaction between syringopicroside and Orfy protein from the perspective of a theoretical calculation. Further tests to verify the combination of the two are necessary.

Interestingly, we found that the expression of an ATP binding cassette transporter, which is a membrane protein belonging to the ABC super family, was enhanced 2.821 times when compared with the control [7]. For biosynthesis of CPS of most bacteria, it is mainly divided into the following three pathways: (1) the Wzy-dependent pathway, (2) the ABC-dependent pathway, and (3) the synthase-dependent pathway [49,50,51]. The above pathway that Orfy protein was involved in belong to the Wzy-dependent pathway [27]. Moreover, the ATP binding cassette transporter, transporting polysaccharide molecules across the membrane is a critical enzyme in the ABC-dependent pathway [52]. Therefore, we speculated that the ABC-dependent pathway was the compensation channel for the synthesis CPS when the Wzy-dependent pathway was inhibited. Further experiments are needed to reveal the relationship between the two pathways.

## 3. Materials and Methods

### 3.1. Bacteria Strain and Cultural Condition

The *S. suis* (ATCC 700794) strain was purchased from American Type Culture Collection (ATCC) and maintained in 50% glycerin at −40 °C. The bacteria were cultured at 37 °C in Todd Hewitt Broth (THB) medium with 5% sheep blood (Feiya Biological Technology Co., Ltd., Nantong, China) for 16 h (Summus Ltd., Harbin, China) when performing the experiments.

### 3.2. Sample Preparation and Reagents

Leaf samples of *S. oblata* were collected in the campus of Northeast Agricultural University (N 45°44′33.64″, E 126°43′22.07″) in Harbin, Heilongjiang Province of China. *S. oblata* raw material was identified by Professor Xiuju Wu (College of Life Sciences, Northeast Agricultural University, Harbin, China). Our herbarium of *S. oblata* with a voucher number (LiYH2017005) has been placed in the Northeast Agricultural University herbarium. HPLC fingerprints of *S. oblata* samples were shown in Figure S1 [53]. Disintegrator (xuman-2000y, Boou, Hefei, China) was used to crush the leaves into powder, which was then filtered using a 50-mesh sieve (Xiangyu Weiye Instrument Equipment Co., Ltd, Beijing, China). Finally, the powder was stored in a vacuum dish after drying in an oven (YHG-9055A, Yaoshi instrument, Shanghai, China) at a temperature of 80 °C for 24 h. Tryptone, yeast, and beef extract were purchased from Aoboxing (Beijing, China). Serum was purchased from Tianhang (Nanjing, China). HPD-500 macroporous resin was obtained from Bohui Biochemical Co., Ltd. (Shenzhen, China). Besides, all other reagents used were of an analytical grade.

### 3.3. Single-Factor Experiments for Extraction of Syringopicroside

The extraction of syringopicroside was performed on an ultrasonic cell breaker (JY92-2D, Ningbo Scientz Technology Co. Ltd., Ningbo, China). 1.0 g of *S. oblata* leaf powder was poured into a beaker and then a certain volume of water was added. The ultrasonic sensor probe reached 1.5 cm below the liquid level. The change range of variables were as follows: 500–900 W (ultrasonic power), 30–90 min (ultrasonic time), 30–70 °C (temperature), and 30:1–70:1 mL/g (liquid-to-solid ratio). When the effect of one variable was investigated, the other variables were fixed at a certain level. Then, the extract solution was filtered, concentrated, and lyophilized. Finally, the samples were measured at 232 nm and the yield was calculated by the following formula [54].

(2) $$Y=\frac{C\times V}{M}$$

where Y represents the yield of syringopicroside, (mg/g). C and V mean the concentration of syringopicroside (mg/mL) and volume (mL) of the sample solution, respectively. M means the mass of the power (g). The standard curve for syringopicroside was: $Y=8.68X-0.0054\left(R^{2}=0.9991\right)$, where Y is the absorbance of the sample and X (mg/mL) is the concentration of syringopicroside.

### 3.4. Experimental Design

Based on the results obtained from the single-factor experiments, four variables including ultrasonic time (*X*_1_), temperature (*X*_2_), a liquid-to-solid ratio (*X*_3_), and ultrasonic power (*X*_4_) and the effect of their interactions on the yield of syringopicroside (*Y*) were investigated by using RSM. The four-factor-three-levels Box-Behnken design (BBD) of RSM was carried out by using Design-expert software (version 8.0), and all the independent variables to be encoded were varied over three levels (−1, 0, 1). The codes and the representative values corresponding to it were shown in Table 4. The process of coding was carried out based on the equation below [55].

(3) $$X_{i}=\frac{x_{i}-x_{0}}{\Delta x}\left(i=1,2,3\right)$$

where $X_{i}$ and $x_{i}$ represent the dimensionless and actual values of the independent variable, i, $x_{0}$ is the actual value in the middle of the domain, and Δx means the increment of $x_{i}$ corresponding to the variation of $X_{i}$ at one unit.

BBD-matrix consisted of 29 groups of experimental runs including 24 factorial points and five central points. Additionally, the details of the experimental conditions and the relevant experimental and predicted values of syringopicroside yield were given in Table 4. The relationship between dependent and independent variables was expressed as a second-order polynomial equation.

(4) $$Y=\beta_{0}+\overset{k}{\underset{i=1}{\sum }}\beta_{i}X_{i}+\overset{k}{\underset{i=1}{\sum }}\beta_{ii}X_{i}^{2}+\overset{k}{\underset{i\leq j}{\sum }}\beta_{ij}X_{i}X_{j}$$

where $\beta_{0}$, $\beta_{i}$, $\beta_{ii}$, and $\beta_{ij}$ represent the regression coefficients for the mean, linear, interaction, and quadratic terms, respectively, and k is the number of independent variables (k = 3 in this investigation). $X_{i}$ and $X_{j}$ are the coded values of the independent variables. Y is the yield of the syringopicroside.

The data from BBD design was analyzed by multiple regressions to fit the functional relationship between factors and response values, and determine the optimum process parameters. The relationship between experimental levels of factors and response value was visualized as surface plots. Analysis of variance (ANOVA) was used to estimate the statistical parameters, and further obtained the regression equations. Finally, under the optimal conditions, experiments were carried out in triplicate to compare the actual value with the predictive value, which, in turn, verified the accuracy and suitability of the model.

### 3.5. Separation of Syringopicroside

The separation method was carried out with a reference to Wei et al. [56]. Sample solution (6.21 mg/mL, 30 mL) was loaded into HPD-500 macroporous resin at the speed of 1.5 mL/min. Subsequently, the distilled water (5 BV) was used to clean the column and remove impurities. Finally, the 40% ethanol solution (5 BV) was used to elute the column at a flow rate of 2 mL/min. The collected liquid was extracted with ethyl acetate for three times in which 50 mL was added each time and extracted for 20 min. Finally, the powder was obtained after concentrating and drying, and then stored in an ultra-low temperature refrigerator for later analysis.

### 3.6. Identification of Syringopicroside

The determination of syringopicroside standard and purified sample was done on an HPLC-MS system. HPLC analysis was performed on an ACQUITY UPLC BEH C18 column (100 mm × 2.1 mm, 1.7 mm) (Waters Technology Co., Ltd., Shanghai, China). In addition, the column temperature was kept at 40 °C. The mobile phase was composed of methanol (A) and water (B) with a volume ratio of 70:30. The flow rate of the mobile phase was 1 mL/min and the injection volume was 5 μL. The mass spectrometer (TripleTOF^®^ 5600+, SCIEX, Shanghai, China) was equipped with an electron spray ionization (ESI) system and controlled by AB analyst software (1.7.1, SCIEX, Shanghai, China). For detailed parameter settings, refer to the previous study [57].

### 3.7. Antibacterial Activity Evaluation of Syringopicroside

#### 3.7.1. MIC Assay

The MIC assay was carried out on the basis of the Clinical and Laboratory Standards Institute (CLSI). The bacteria solution (5 × 10^5^ CFU/mL) was added to a 96-well plate with a pipette gun, smf 315 μL of the bacteria solution was added to the first row of the first well. Furthermore, 175 μL of the bacteria solution was added to the other nine wells, and then 35 μL of a drug in a moderate concentration (10.24 mg/mL) was added to the first well. After blending, the mixture (175 μL) was removed from the first well and added into the second well, and then micro-dilution was done in turn. When the operation was finished in the 10th well, 175 μL of liquid was drawn from the last hole. Then, 175 μL of methanol was added to the 11th well as solvent control and 175 μL of medium was added to the 12th well as a negative control. Moreover, the wells with bacterial liquid and no drugs were used as a positive control. The MIC value of the drug to bacteria was observed by the naked eye after 20–24 h of incubation at 37 °C.

#### 3.7.2. Biofilm Assay

A biofilm assay was carried out according to the method described by Wang et al. [48]. Based on the result of the MIC assay, the culture time was prolonged to 72 h. After forming the biofilm, the 96-well plate was placed upside down on the filter paper to remove the bacterial solution, and then the 96-well plate was dipped into the phosphoric acid buffer solution (PBS, pH 6.8) to remove the unattached bacteria. For a dyeing process, the excess liquid was removed by inverting the 96-well plate onto the filter paper, and then 200 μL of 99% methanol was added into each well and let the 96 well plate stand for 30 min. Second, 200 μL of 1% crystal violet solution was dyed for 30 min, and rinsed with running water to be air-dried. Third, 200 μL of 33% glacial acetic acid was added and placed in oscillators for 10 min to release crystal violet in biofilm. The optical density (OD) value was measured at 595 nm by an enzyme-labeled analyzer.

### 3.8. SEM Observation

Two pieces of sterilized frosted glass were first put into a 6-well microplate, and then the biofilm culture was conducted according to the method mentioned above. One well without adding syringopicroside was used as a control group, and the other well with syringopicroside (1/2 MIC) was used as an experimental group. After the formation of biofilm by *S. suis* on frosted glass, the culture medium was discarded and 2.5% of glutaraldehyde was added. Then the mixture was placed in a refrigerator (4 °C) for 1.5 h. At the rinsing stage, 0.1 mol/L of PBS (pH 6.8) was applied three times at an interval of 10 min each. In order to achieve the purpose of dehydration, 70%, 80%, and 90% ethanol were added to the experimental unit, respectively, and then 100% ethanol was added thrice at an interval of 10 min each. Next, a mixture of *tert*-butyl alcohol solution and anhydrous ethanol (1:1) and a pure *tert*-butyl alcohol solution were sequentially added at an interval of 15 min. The glass was stored in a refrigerator at −20 °C, and then it was dried in a Freeze dryer (ES-2030, HITACHI, Tokyo, Japan) for 4 h. The glass was fixed on the sample table with conductive tape and coated the gold film with a thickness of 100–150 Å using an ion sputtering coater (E1010, HITACHI, Tokyo, Japan). Finally, the observation of *S. suis* biofilm was carried out under an electron microscopy (S-3400N, HITACHI, Tokyo, Japan), which is referred to in a previous study [58].

### 3.9. Molecular Docking

The three-dimensional structure of an Orfy crystal structure had to be built because it had not been reported before. Originally, the primary structure of Orfy was obtained from Uniprot (https://www.uniprot.org/ accessed on 26 February 2021) [59]. The protein structure was modeled by ab initio modeling through Robetta sever (http://robetta.bakerlab.org/ accessed on 26 February 2021) [60]. The syringopicroside structure was obtained from Pubchem (https://pubchem.ncbi.nlm.nih.gov accessed on 26 February 2021) [61]. Second, the sitemap was performed to predict the binding site of Orfy protein and the site with the highest score was selected. The coordinate of the binding site was X: 21.04, Y: −2.52, Z: −12.24. The size of the site was 15 Å. Depending on CHARMm force field, CDOCKER (Chuangteng Technology Co., Ltd., Beijing, China) was applied to molecular docking. Finally, the docking results were arranged according to the scoring function, and the lowest energy conformation was selected for further study. The complex structure was analyzed and visualized by PyMoL v2.0.6. software [62].

### 3.10. Statistical Analysis

All experiments were carried out for three times and the results were represented by mean ± standard deviation. Statistical analysis was performed using SPSS 19.0. *p* < 0.05 was considered as statistically significant.

## 4. Conclusions

In this study, the optimum UAE conditions for syringopicroside extraction from *Syringa oblata* Lindl leaves were obtained through RSM. RSM analysis indicated that the liquid-to-solid ratio significantly affected the syringopicroside yield. Syringopicroside showed good performance in inhibiting *S. suis* and the biofilm formation. Moreover, syringopicroside exhibited a good affinity with Orfy protein of *S. suis* by hydrogen bonds, hydrophobic interaction, and π-π stacking. These results showed that syringopicroside may have a potential to be used as alternatives to synthetic antimicrobial agents in the food industry and antibiotics in the treatment of diseases caused by *S. suis*. Further work is needed to investigate the bioavailability of syringopicroside in vivo to understand the underlying molecular mechanism of actions.

## Figures and Tables

**Table 1:** The experimental design, actual value, and the predictive value by the response surface methodology (RSM) model.

Run	Variable Levels				Y (mg/g)	
	*X*_1_ (min)	*X*_2_ (°C)	*X*_3_ (mL/g)	*X*_4_ (W)	Observed	Predicted
1	−1	0	0	−1	2.29 ± 0.13	2.29
2	1	0	0	1	2.82 ± 0.11	2.82
3	0	1	0	−1	2.57 ± 0.09	2.59
4	1	0	0	−1	2.46 ± 0.12	2.49
5	1	−1	0	0	2.79 ± 0.03	2.86
6	0	0	0	0	3.08 ± 0.06	3.02
7	0	−1	0	1	2.92 ± 0.04	2.90
8	0	0	1	1	2.85 ± 0.06	2.86
9	−1	1	0	0	2.81 ± 0.05	2.75
10	−1	0	−1	0	2.04 ± 0.10	1.96
11	−1	0	0	1	2.73 ± 0.07	2.69
12	0	−1	−1	0	2.26 ± 0.03	2.15
13	−1	−1	0	0	2.26 ± 0.07	2.47
14	1	0	1	0	2.71 ± 0.11	2.79
15	0	0	0	0	2.97 ± 0.09	3.02
16	1	1	0	0	2.89 ± 0.05	2.69
17	0	1	−1	0	2.05 ± 0.06	2.18
18	0	0	1	−1	2.59 ± 0.06	2.55
19	0	−1	0	−1	2.31 ± 0.03	2.30
20	0	0	0	0	2.93 ± 0.04	3.02
21	0	1	1	0	2.75 ± 0.05	2.86
22	0	0	0	0	2.95 ± 0.05	3.02
23	0	−1	1	0	2.62 ± 0.08	2.58
24	0	0	−1	−1	1.83 ± 0.08	1.83
25	0	0	0	0	3.16 ± 0.11	3.02
26	0	0	−1	1	2.21 ± 0.10	2.26
27	0	1	0	1	2.71 ± 0.06	2.72
28	−1	0	1	0	2.51 ± 0.04	2.56
29	1	0	−1	0	2.24 ± 0.07	2.26

Note: Y represents the yield of syringopicroside.

**Table 2:** ANOVA of the regression model for the prediction of syringopicroside yield.

Source	Sum of Squares	df	Mean Square	*F* Value	*p*-Value
Model	3.12	14	0.22	18.73	<0.0001 **
*X*_1_	0.13	1	0.13	11.30	0.0047 **
*X*_2_	0.032	1	0.032	2.69	0.1230
*X*_3_	0.96	1	0.96	81.01	<0.0001 **
*X*_4_	0.40	1	0.40	33.61	<0.0001 **
*X*_1_ *X*_2_	0.051	1	0.051	4.26	0.0581
*X*_1_ *X*_3_	0.000	1	0.000	0.000	1.0000
*X*_1_ *X*_4_	1.600 × 10^−3^	1	1.600 × 10^−3^	0.13	0.7193
*X*_2_ *X*_3_	0.029	1	0.029	2.43	0.1413
*X*_2_ *X*_4_	0.055	1	0.055	4.64	0.0491 *
*X*_3_ *X*_4_	3.600 × 10^−3^	1	3.600 × 10^−3^	0.30	0.5909
*X*_1_^2^	0.26	1	0.26	21.69	0.0004 **
*X*_2_^2^	0.15	1	0.15	12.38	0.0034 **
*X*_3_^2^	1.23	1	1.23	103.53	<0.0001 **
*X*_4_^2^	0.35	1	0.35	29.34	<0.0001 **
Residual	0.17	14	0.012	-	-
Lack-of-fit	0.13	10	0.013	1.32	0.4237
Pure error	0.039	4	9.670 × 10^−3^	-	-
Cor total	3.28	28	-	-	-
*C.V*%	-	4.20		-	-
*R* ^2^	-	0.9493		-	-
*Adj-R* ^2^	-	0.8986		-	-

Note: **: Highly significant coefficient (*p* < 0.01), *: Significant coefficient (*p* < 0.05).

**Table 3:** The specific interaction forms between syringopicroside and the Orfy protein.

Donor Force	Receptor Force	Interaction Type
syringopicroside: OH	ASN54: O	Hydrogen bond
syringopicroside: OH	GLY63: O	Hydrogen bond
LYS25: OH	syringopicroside: O	Hydrogen bond
LYS386: OH	syringopicroside: O	Hydrogen bond
TYR57	syringopicroside	π-π stacking

**Table 4:** Design factor and horizontal coding of response surface methodology.

Coded Symbol	Independent Variable	Units	Levels		
			1	0	−1
*X*_1_	Ultrasonic time	min	75	60	45
*X*_2_	Temperature	°C	70	60	50
*X*_3_	Liquid-to-solid ratio	mL/g	70:1	60:1	50:1
*X*_4_	Ultrasonic power	W	900	800	700

## Data Availability

All data included in this study are available upon request by contact with the corresponding author.

## Supplementary Materials

The following are available online, Figure S1: Chromatographic fingerprints for reference (A) and all the *Syringa oblata* Lindl sample (B), Figure S2: Quality assessment of optimized Orfy protein.

## Sample Availability

Samples of the compounds for this experiment are available upon request by contact with the corresponding author.
