# Supplementary material for: Effect of Syringopicroside Extracted from Syringa oblata Lindl on the Biofilm Formation of Streptococcus suis
Source: Molecules. 2021 Feb 27;26(5):1295. doi: 10.3390/molecules26051295 (PMC7957517; doi:10.3390/molecules26051295)
Supplement: Supplementary file 1 [file molecules-26-01295-s001.pdf]

Supplementary materials

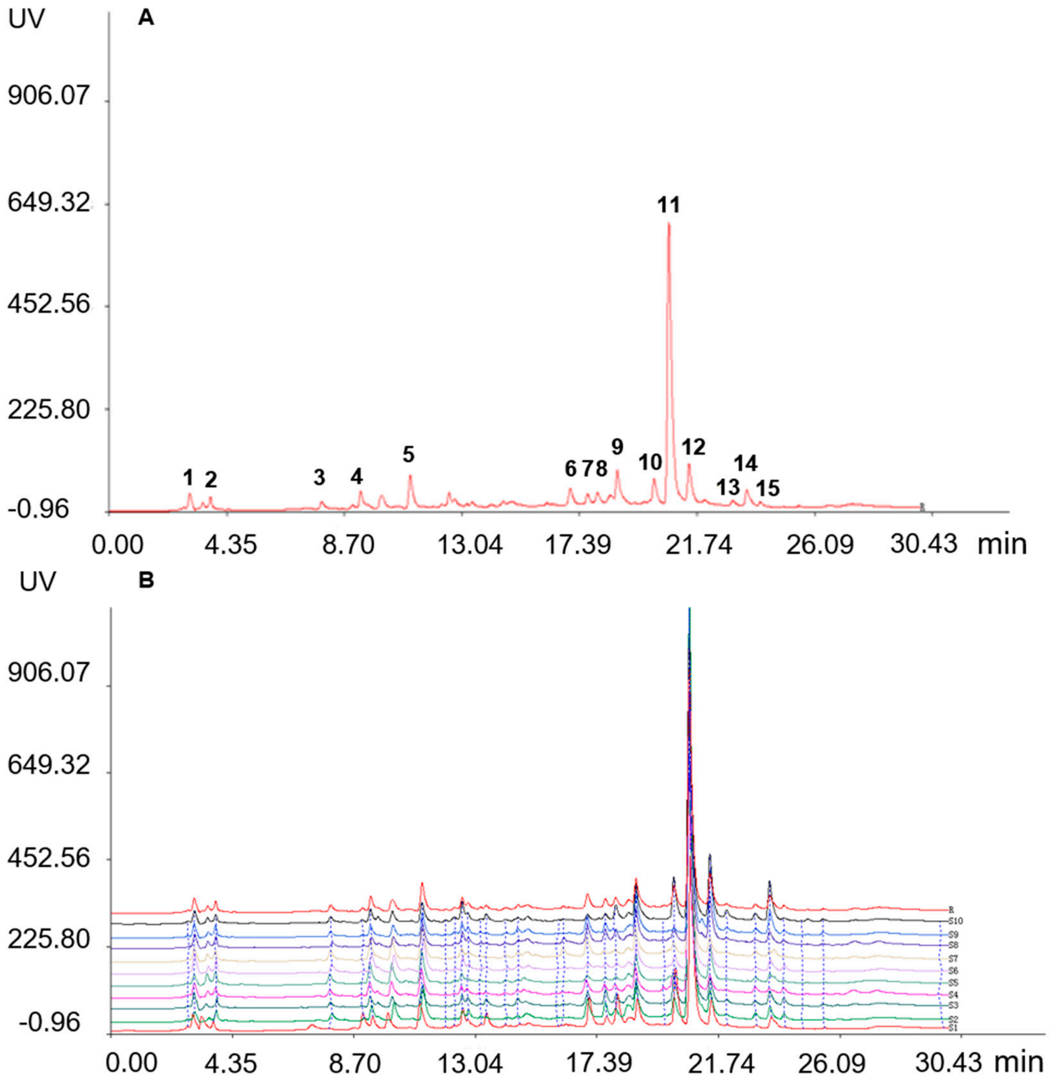

Figure S1 Chromatographic fingerprints for reference (A) and all the *Syringa oblata* Lindl sample (B). The number 7 represents rutin, the number 8 represents demethyloleuropein, the number 10 represents syringopicroside, and the number 12 represents oleuropein, in (A).

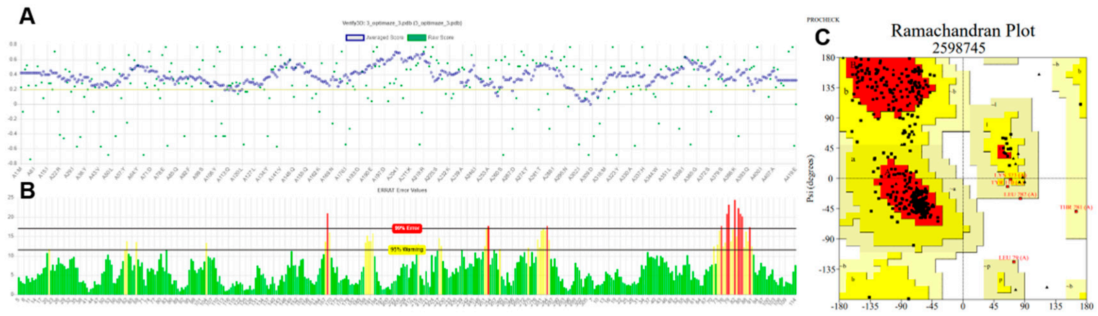

Figure S2 Quality assessment of optimized Orfy protein. The graphic of Verify 3D (A). The evaluation of ERRAT (B). Ramchandran plot of Orfy protein (C).
